# Clinical and Radiographic Features of Peri‐Implant Medication‐Related Osteonecrosis of the Jaw: A Retrospective Study

**DOI:** 10.1111/cid.13412

**Published:** 2024-11-06

**Authors:** Hyeon‐Gyu Jo, Wonse Park, In‐Ho Cha, Young‐Soo Jung, Da Yun Lee, Jun‐Young Kim

**Affiliations:** ^1^ Department of Oral and Maxillofacial Surgery Yonsei University College of Dentistry Seoul South Korea; ^2^ Department of Advanced General Dentistry Yonsei University College of Dentistry Seoul South Korea; ^3^ Department of Statistics University of Seoul Seoul South Korea

**Keywords:** anti‐resorptive drugs, dental implant, jaw, osteonecrosis

## Abstract

**Introduction:**

This study aimed to analyze the factors affecting the occurrence of peri‐implant medication‐related osteonecrosis of the jaw (PI‐MRONJ) in patients using anti‐resorptive drugs (ARDs) on different implant position, inclinations, and types of prosthesis.

**Methods:**

The data of 75 patients with bone necrosis that progressed around the implant between 2018 and 2022 were retrospectively examined to identify the factors influencing PI‐MRONJ. Data, including patient demographics (age, sex, smoking status, concomitant disease, time of ARD therapy, dose of ARDs, and parafunctional habits) and implant‐specific information (type of prosthesis, angle of insertion), were extracted from medical and dental records.

**Results:**

Tilted implants with an angle ≥ 5.1° relative to the occlusal plane of the prosthesis had a stronger association with PI‐MRONJ in comparison to non‐tilted implants (inclination was < 5°). Additionally, the boundary of the area of osteonecrosis around the fixture was larger for the splinted implant‐supported crowns than for the single implant supported crowns).

**Conclusion:**

In patients taking anti‐resorptive medications the inclination of the implant was associated with the occurrence of PI‐MRONJ. Further studies are required to confirm the clinical findings.

## 1 Introduction

The pathophysiology of peri‐implant (PI)‐medication‐related osteonecrosis of the jaw (MRONJ) has not yet been fully elucidated; moreover, it is even less well understood than that of MRONJ and also appears to be multifactorial. Nevertheless, PI‐MRONJ may be associated with the same risk factors, such as history of antiresorptive drugs (ARDs), age, sex, corticosteroid therapy, route of administration, and presence or absence of a systemic disease [1]. ARDs are used for the treatment of bone diseases, such as osteoporosis, cancer with bone metastasis, multiple myeloma, and osteogenesis imperfect. However, the influence of ARDs on failure after implant placement and failure of existing implants has not been elucidated [2].

The lack of clear guidelines on the association between implants and ARDs could pose challenges in decision‐making for implant placement in patients receiving osteoporosis medication. According to several studies, ARD therapy demonstrated no statistically significant effect on implant survival or success; thus, patients with osteoporosis are not absolutely contraindicated for implant treatment [3].

The currently known major potential causes of MRONJ range from surgical factors, such as tooth extraction, implant placement, and surgical periodontal techniques, to local factors, such as properties of the upper and lower jaw bones and the presence or absence of oral lesions [4]. Systemic factors such as a history of smoking and/or steroid use, age, and presence or absence of systemic diseases are also worth considering [5].

The prognosis of implants in patients receiving ARDs remains unclear; however, previous studies have suggested implant placement and the presence of the implant itself as risk factors leading to osteonecrosis in patients receiving these antiresorptive drugs [6].

A previous study demonstrated that occlusal force resulted in loading on the adjacent bone during mastication with the implant, and the range and magnitude of the loading varied according to the implant inclination. Additionally, it was confirmed that overload can be applied to certain alveolar bone parts [7].

Therefore, this study aimed to investigate osteonecrosis affecting the area around the implant in patients using ARDs and analyze clinical features of the lesions, such as implant position and inclination. New factors were determined in association with the existing risk factors.

## 2 Materials and Methods

### 2.1 Study Design and Sample Selection

This retrospective study involved 75 patients who were using medications associated with MRONJ, exhibited osteonecrosis in the jawbone around the implant fixture, and were admitted to the Department of Oral and Maxillofacial Surgery and Department of Advanced General Dentistry at Yonsei University Dental Hospital from January 2018 to December 2022.

We first checked electronic medical records for patients who had a history of using antiresorptive drugs at our hospital and were diagnosed with MRONJ, and x‐ray images of patients with exclusive osteonecrosis around the implant fixture were checked. Among these patients, those who had undergone CT scans for more than a year after implant placement and showed a condition in which osteonecrosis was observed around the implant unlike peri‐implantitis, were included. These patients were screened using electronic medical records that contained radiographs, outpatient records, and progress notes. Information was classified based on criteria, including sex, age, presence or absence of a systemic disease other than osteoporosis, parafunctional habits, history of using ARDs, type, and dosage of currently administered osteoporosis‐related drugs or ARDs, site of osteomyelitis, area of the lesion, and type of surgery.

The study protocol was approved by the Institutional Research Ethics Committee of the Yonsei University College of Dentistry (IRB No. 2–2023‐0022) and was conducted in accordance with the STROBE guidelines for cohort studies. Written or verbal informed consent was not obtained from any of the participants because the IRB waived the need for individual informed consent, as this study had a noninterventional retrospective design. Additionally, all x‐ray images were anonymized to ensure confidentiality.

### 2.2 Definition of Peri‐Implant MRONJ

**FIGURE 1:**
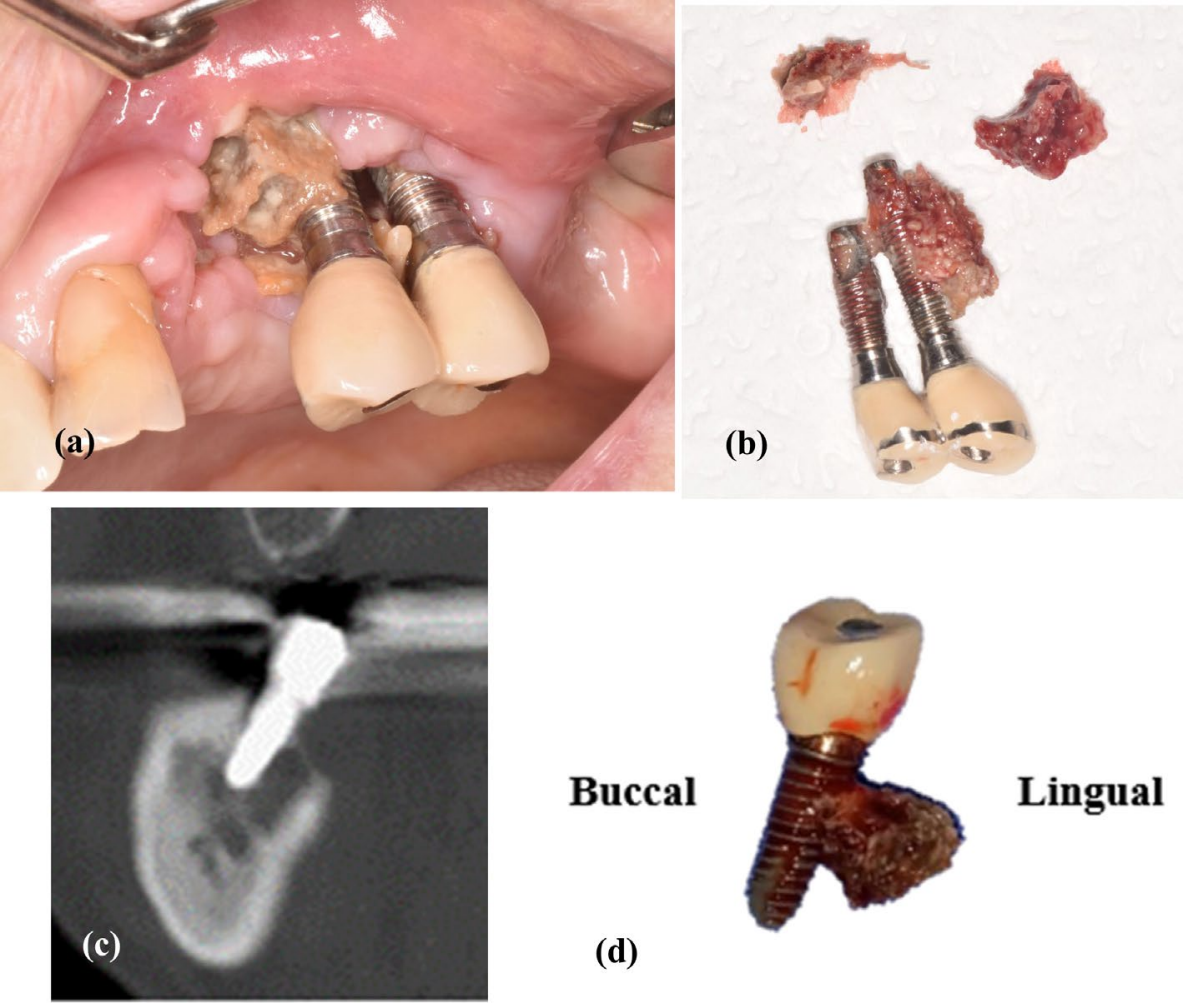
Clinical aspect of the bone necrosis areas affecting the implant region. (a, b) Representative clinical photographs of peri‐implant medication‐related osteonecrosis of the jaw. (c) Preoperative radiographic view with lingual tilted inclination. (d) Postoperative image showing implant fixture with a sequestrum.

The diagnosis of MRONJ was confirmed based on the following American Association of Oral and Maxillofacial Surgeons diagnostic criteria: (1) current or previous treatment with antiresorptive therapy alone or in combination with immune modulators or antiangiogenic medications; (2) exposed bone or bone that can be probed through an intraoral or extraoral fistula(e) in the maxillofacial region that has persisted for more than 8 weeks; and (3) no history of radiation therapy or metastatic disease to the jaw [8]. All the patients had osseointegrated implants for more than 1 year and showed characteristic osteonecrosis morphology in the area around the implant. PI‐MRONJ, which occurs early after implant placement, was thought to be influenced by other variables, such as unhealed sockets; thus, only those lesions that occurred after a sufficient period of time after implantation were considered. PI‐MRONJ was determined based on the following criteria for patients showing potential MRONJ: (1) osteonecrosis in the area around the implant fixture, (2) extensive osteonecrosis with or without sequestrum, and (3) sequestrum with bone contact in the area around the implant fixture (Figure 1) [6].

However, beyond the clinical characteristics of implant‐related osteonecrosis around the implant, a clear definition of PI‐MRONJ is currently lacking. In this study, patients with PI‐MRONJ were clinically classified according to the aforementioned conditions. Some patients also had MRONJ related to an implant site where adjacent teeth had been extracted, referred to as PI‐MRONJ Type II.

**FIGURE 2:**
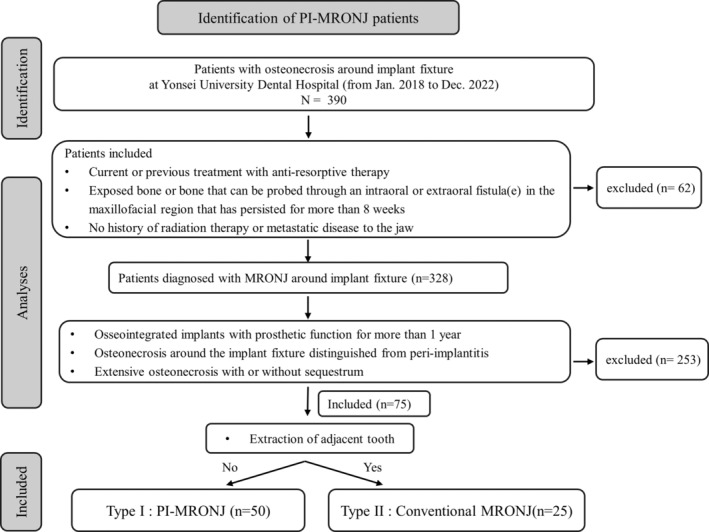
Study flow chart. MRONJ, medication‐related osteonecrosis of the jaw.

Therefore, we observed the following two main patterns of bone destruction around the implant: Type I (PI‐MRONJ) and Type II (conventional MRONJ). In this study, patients with type II destruction included those with peri‐implant osteonecrosis extending from the extraction of adjacent teeth (*n* = 25) (Figure 2).

### 2.3 Patient‐ and Implant‐Related Variables

The following patient‐related variables were obtained from the records: age, sex, type of edentulous opposing arch (complete/partial), smoking status (smoker, former smoker, nonsmoker), steroid use, underlying disease, parafunctional habits, time of ARD therapy, MRONJ incidence (solitary/multiple), and history of periodontitis.

Patients receiving low‐dose ARDs (e.g., for osteoporosis) generally have a low risk of developing MRONJ. Conversely, high‐risk groups include individuals taking high doses of ARDs for skeletal events, metastasis prevention (e.g., in breast or prostate cancer), and multiple myeloma treatment. Thus, in this study, we also categorized the patients based on the typical dosages for both low and high‐dose ARDs [2].

PI‐MRONJ‐associated implants were compared to normally functioning implants in other quadrants. Implant‐related variables included implant position (maxilla/mandible), type of prosthesis, type of opposing occlusion, implant crestal module design (bone/tissue level), implant system (diameter/length), and implant inclination. In this study, dental panoramic radiographs and computed tomography (CT) images were analyzed to determine implant inclination and the boundary of osteonecrosis around the fixture. Differences between the normal implant area and the lesion‐affected area were examined in each patient. The angle between a horizontal line on the occlusal plane of the prosthesis and the inclination of the implant placement was used for categorization [1, 9].

**FIGURE 3:**
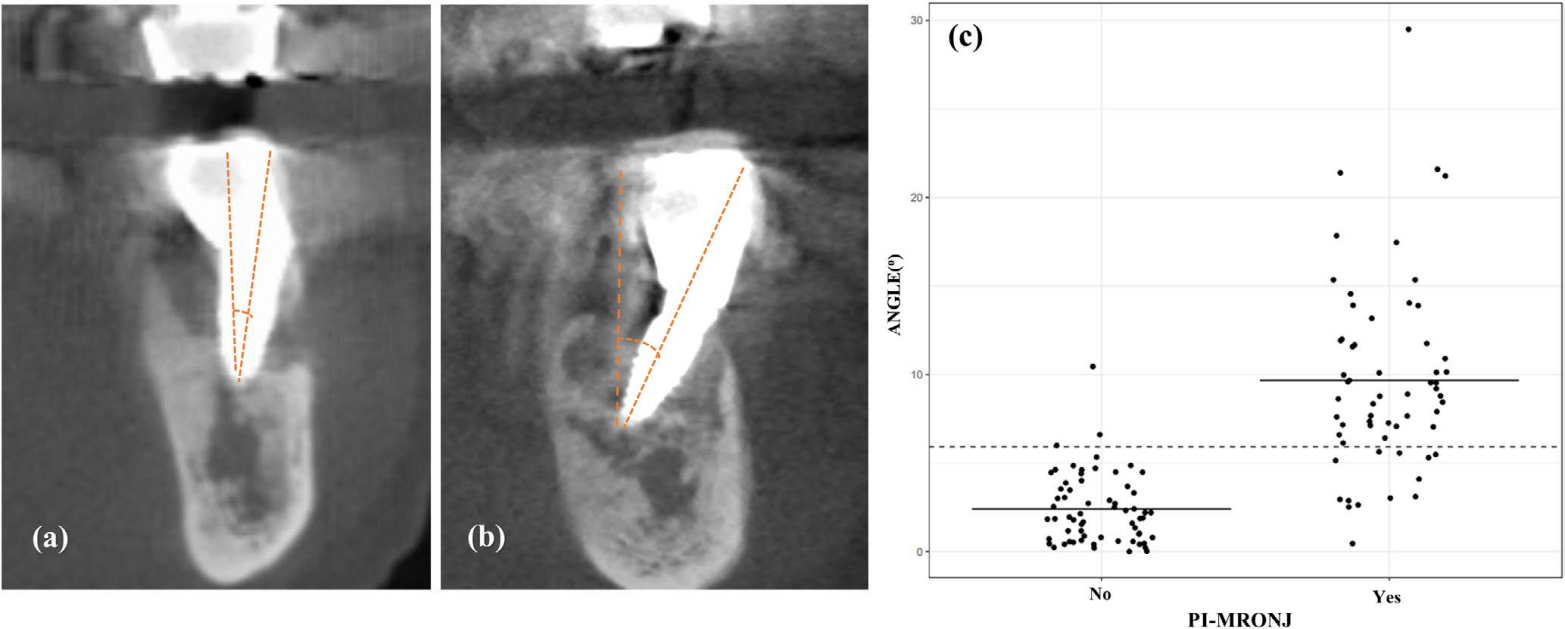
Representative cone‐beam computed tomography of two different case. (a) Implant with moderate implant inclination (5.1^o^–14.9^o^). (b) Implant with severe implant inclination (≥ 15^o^). (c) The Scatter plot shows the distribution of the normal implants and those affected by peri‐implant medication‐related osteonecrosis of the jaw.

In the comparison group not affected by PI‐MRONJ, implant inclination > 15° was not present, and most of the implants (56/64, 87.5%) tended to be distributed at an inclination within 5°. Implant inclination was categorized as acceptable (≤ 5°), moderate (5.1°–14.9°), and severe (≥ 15°) (Figure 3). As the occlusal plane and inclination could not be determined for the anterior region, only cases of PI‐MRONJ in the posterior region were considered.

### 2.4 Statistical Analysis

To determine whether various implant‐related factors had significant effects on PI‐MRONJ, a Generalized Estimating Equations (GEE) model was applied using R (version 4.2.2; R Foundation for Statistical Computing, Vienna, Austria). For each implant‐related factor (implant location, type of prosthesis, type of opposing teeth, implant crestal module design, diameter, length, and inclination), odds ratios (ORs), 95% confidence intervals (CIs), and *p*‐values were calculated through GEE. Furthermore, the ORs, 95% CIs, and *p*‐values were obtained through generalized estimating equations for all factors. To compare the mean size per type of prosthesis (single, splinted, and bridge), analysis of variance (ANOVA) was performed using SAS version 9.4 (SAS Institute, Cary, NC). One‐way ANOVA with Bonferroni's post hoc tests was used to compare variations in the mean size between the three prosthesis types.

## 3 Results

### 3.1 Demographic Data

The data of 75 patients (10 male patients [13.3%] and 65 female patients [86.7%]) were analyzed in this study. The mean age of the patients was 76.3 ± 9.2 years, indicating that most patients were older adults. Overall, 65 patients were receiving medication for osteoporosis, and the remaining 10 were receiving medication for tumors (owing to a history of breast cancer [*n* = 4], prostate cancer [*n* = 3], or multiple myeloma [*n* = 3]).

Patients with oncological issues who were affected by implant‐related bone necrosis were treated with high‐dose ARDs. Four patients in the group using high doses had multiple lesions due to oncological problems, and six patients had solitary lesions.

Conversely, in the low‐dose group, 6 patients had multiple lesions; however, 59 patients had solitary lesions, which proportionally tended to result in more multiple lesions in the high‐dose group. Moreover, in the group using high‐dose ARDs, bone destruction around the implant ranged approximately 23.92 ± 10.67 mm, whereas in the low‐dose ARDs groups, bone destruction was observed in a relatively small range of 21.08 ± 9.57 mm (*p = 0.3493*). Nevertheless, no statistical significance was observed owing to the small number of patients.

Patients usually had solitary lesions (65/75, 86.7%). Among the 65 patients with solitary lesions, 40 patients with a history of ARDs use after implantation reported the onset of PI‐MRONJ after a mean duration of 2.64 years. In contrast, in 25 patients, PI‐MRONJ developed at approximately 2.52 years after implantation despite receiving ARDs. Parafunctional habits, such as bruxism, were noted in two patients. In most patients, a single quadrant of the mandible in the area around the implant was affected; however, 10 patients had multiple (more than 2 quadrants) peri‐implant lesions (Table 1).

**TABLE 1:** Patient demographics.

Patients^a^ (*n* = 75), *n* (%)		
Sex		
Female/Male		65 (86.7%)/10 (13.3%)
Smoking status		
Non‐smoker/Smoker		71 (94.7%)/4 (5.3%)
Diabetes mellitus		
No/Yes		55 (73.3%)/20 (26.7%)
Parafunctional habits (e.g., Clenching, bruxism)		
No/Yes		73 (97.3%)/2 (2.7%)
Steroid use		
No/Yes		63 (84.0%)/12 (16.0%)
History of periodontitis		
No/Present		41 (54.7%)/34 (45.3%)
Type of edentulous opposing arch		
Partial/Complete		70 (93.3%)/5 (6.78%)
Time of ARD therapy (year)		
≤ 2		29 (38.7%)
> 2, ≤ 4		20 (26.7%)
> 4		26 (34.6%)
Mean ± SD		3.89 ± 2.77
Underlying disease for ARD use		
Osteoporosis	Low dose ARDs	65 (86.7%)
Oncology^b^	High dose ARDs	10 (13.3%)
ARD (dose, route)		
Ibandronate (low dose, 70 mg/week per os.)		17 (22.7%)
Alendronate (low dose, 70 mg/week per os.)		17 (22.7%)
Pamidronate (low dose, 30 mg/3 months i.v.)		2 (2.7%)
Risedrnoate (low dose, 35 mg/week per os.)		6 (8.0%)
Zoledronate	low dose, 5 mg/year i.v.	5 (6.7%)
	high dose, 4 mg/3 ~ 4 weeks i.v.	6 (8.0%)
Denosumab	low dose, 60 mg/6 months s.c.	18 (24.0%)
	high‐dose, 120 mg/3 ~ 4 weeks s.c.	4 (5.3%)
MRONJ occurrence		
Solitary	low dose ARDs	59 (78.7%)
	high dose ARDs	6 (8.0%)
Multi‐location	low dose ARDs	6 (8.0%)
	high dose ARDs	4 (5.3%)

^a^ Mean ± SD: age, 76.3 ± 9.2 year; 2.8 implants per patient.

^b^ For example, Breast cancer, Prostate cancer, Multiple myeloma.

Abberviation: ARD, antiresorptive drug; i.v., intravenous; MRONJ, medication‐related osteonecrosis of the jaw; s.c., subcutaneous.

Among the 178 implants osseointegrated for more than 1 year in the posterior region of 75 patients, most (94.38%) showed an inclination ranging from acceptable to moderate (0°–14.9°), and the remaining 10 showed a tendency for severe (≥ 15^o^) tilting (Table 2).

**TABLE 2:** Implant information.

	Implants (*n* = 178), *n* (%)	
	Type I: PI‐MRONJ (*n* = 124), *n*(%)	Type II: Conventional MRONJ (*n* = 54), *n*(%)
Implant location (Jaw)		
Maxilla	46 (37.10%)	18 (33.33%)
Mandible	78 (60.67%)	36 (66.67%)
Type of prosthesis		
Bridge*	27 (21.77%)	7 (12.96%)
Single	35 (28.23%)	19 (35.19%)
Splinted*	62 (50.00%)	28 (51.85%)
Type of opposing occlusion		
Implant	56 (45.16%)	30 (55.56%)
Teeth	39 (31.45%)	16 (29.63%)
Mixed	23 (18.55%)	8 (14.81%)
Denture	6 (4.84%)	0
Implant crestal module design		
Bone level	94 (75.81%)	42 (77.78%)
Tissue level	30 (24.19%)	12 (22.22%)
Diameter (mm)		
Regular (≤ 4.9)	95 (76.61%)	41 (75.93%)
Wide (5.0 ≥ )	29 (23.39%)	13 (24.07%)
Length (mm)		
Short (≤ 8.0)	3 (2.42%)	4 (7.41%)
Regular (8.1–11.9)	89 (71.77%)	38 (70.37%)
Long (12.0 ≥)	32 (25.81%)	12 (22.22%)
Inclination (Implant angle)		
Acceptable (≤ 5^o^)	64 (51.62%)	36 (66.67%)
Moderate (5.1^o^–14.9^o^)	52 (41.94%)	16 (29.63%)
Severe tilting (15^o^≥)	8 (6.45%)	2 (3.70%)

* Implant‐supported multi‐unit prosthesis (e.g., splinted, bridge prostheses).

### 3.2 Risk Factors Between the Implant Levels

In the univariate analysis, PI‐MRONJ appeared to be correlated with the implant location and inclination; however, in the multivariate analysis, the correlation with the implant location was not significant. Based on the multivariate analysis, implants with an inclination of ≥ 5.1° were significantly more associated with the occurrence of PI‐MRONJ than those with an inclination of < 5° (*p < 0.001*). All severely tilting implants (*n* = 8) were affected by PI‐MRONJ (Table 3).

**TABLE 3:** Generalized estimating equations analysis of risk factors for prevalence of PI‐MRONJ.

	Implants at Risk (Normal vs. PI‐MRONJ)	Univariate analysis			Multivariate analysis		
		Estimates	95% CI	*p*	Estimates	95% CI	*p*
Implant location							
Maxilla	30 vs. 16	Ref	Ref	Ref	Ref	Ref	Ref
Mandible	34 vs. 44	0.2163	0.01 ~ 0.42	**0.0359**	0.5164	−0.58 ~ 1.61	0.3558
Type of prosthesis							
Single	18 vs. 17	Ref	Ref	Ref	Ref	Ref	Ref
Splinted	34 vs. 28	−0.0341	−0.31 ~ 0.24	0.8056	0.1631	−1.76 ~ 2.09	0.8679
Bridge	12 vs. 15	0.0698	−0.24 ~ 0.37	0.6532	0.3017	−1.85 ~ 2.46	0.7840
Type of opposing occlusion							
Implant	34 vs. 22	Ref	Ref	Ref	Ref	Ref	Ref
Teeth	18 vs. 21	0.1456	−0.11 ~ 0.40	0.2624	0.1166	−1.25 ~ 1.48	0.8671
Mixed	10 vs. 13	0.1724	−0.11 ~ 0.45	0.2238	1.5484	−0.22 ~ 3.32	0.0859
Denture	2 vs. 4	0.2738	−0.17 ~ 0.72	0.2308	1.0389	−1.85 ~ 3.93	0.4809
Implant crestal module design							
Bone level	48 vs. 46	Ref	Ref	Ref	Ref	Ref	Ref
Tissue level	16 vs. 14	−0.0227	−0.27 ~ 0.22	0.8560	0.7454	−0.84 ~ 2.33	0.3560
Diameter (mm)							
Regular (4.0–4.9)	49 vs. 46	Ref	Ref	Ref	Ref	Ref	Ref
Wide (5.0 ≥)	15 vs. 14	−0.0015	−0.24 ~ 0.24	0.9906	−0.0299	−1.53 ~ 1.47	0.9688
Length (mm)							
Short (≤ 8.0)	1 vs. 2	Ref	Ref	Ref	Ref	Ref	Ref
Regular (8.1–11.9)	46 vs. 43	−0.1835	−0.73 ~ 0.36	0.5078	−3.0007	−6.28 ~ 0.28	0.0728
Long (12.0 ≥)	17 vs. 15	−0.1979	−0.78 ~ 0.38	0.5039	−3.2256	−7.09 ~ 0.64	0.1021
Inclination (Implant angle)							
Acceptable (≤ 5^o^)	56 vs. 8	Ref	Ref	Ref	Ref	Ref	Ref
Moderate (5.1^o^−14.9^o^)	8 vs. 44	0.7212	0.56 ~ 0.88	**< 0.001**	4.2164	2.77 ~ 5.67	**< 0.001**
Severe (15^o^≥)	0 vs. 8	0.8750	0.79 ~ 0.97	**< 0.001**	45.0527	43.76 ~ 46.34	**< 0.001**

Abberivation: CI, confidence interval; PI‐MRONJ, peri‐implant medication‐related osteonecrosis of the jaw; Significant results are presented in bold.

### 3.3 Bone Loss Tendency Between Groups (Type I vs. Type II)

The patterns of bone destruction observed around the implant were categorized into two types. In both groups, clinical features such as the presence of bone necrosis around the implant were in line with the diagnosis flow chart presented earlier. However, some differences were identified.

**FIGURE 4:**
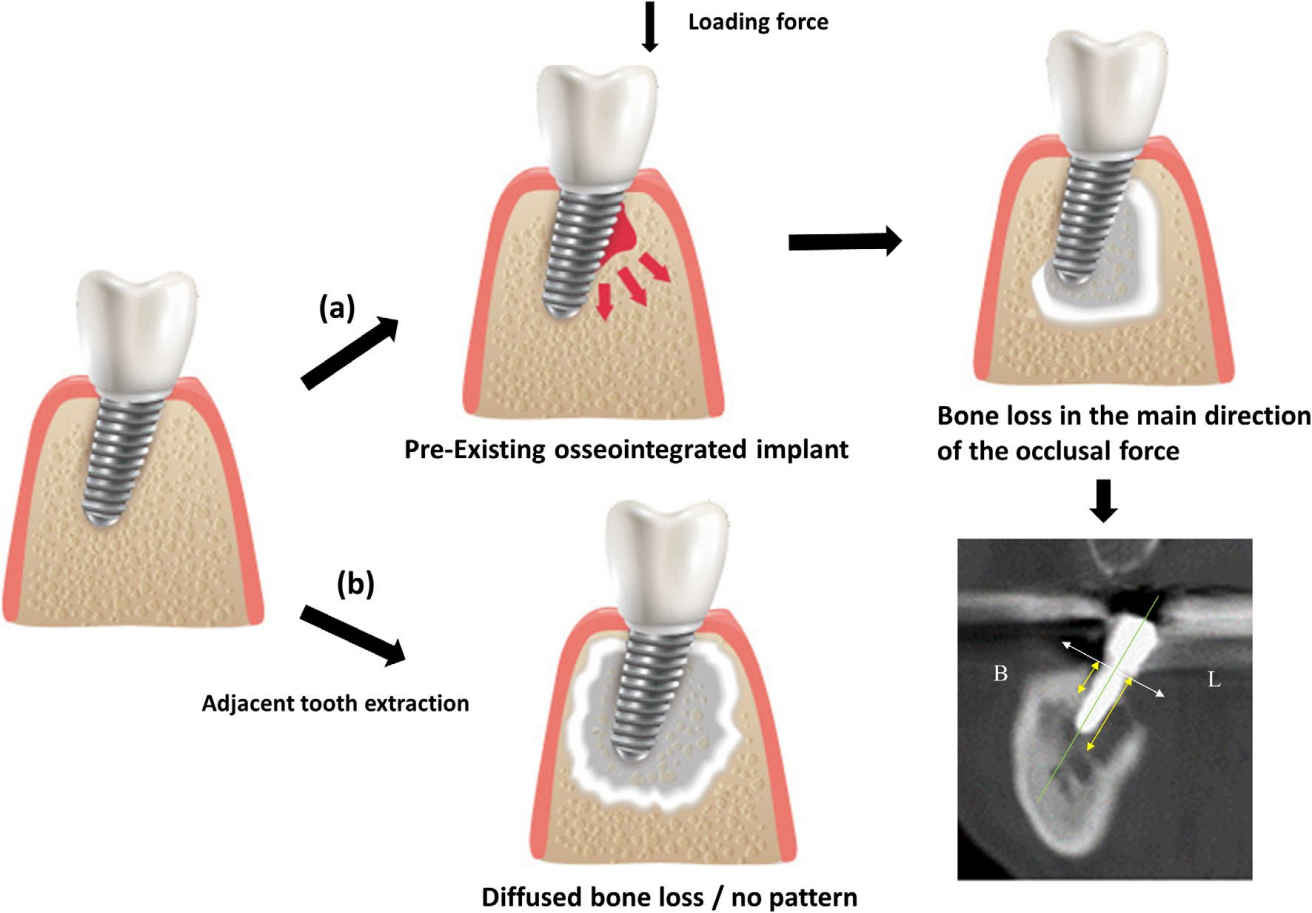
Illustration of two types of implant‐related osteonecrosis progression around the implant. (a) peri‐implant medication‐related osteonecrosis of the jaw (PI‐MRONJ), bone loss predominantly occurring in specific areas around the implant. (b) Conventional MRONJ, diffused bone loss pattern without a notable trend.

For patients in the Type I group, the findings suggested that the bone loss pattern predominantly occurred in areas around the implant, with a gradual spread of the lesion to the surrounding area. When the buccal and lingual area bone loss measurements were performed on 48 implants, a difference of 9.33 ± 4.05 mm was observed on the affected side and 5.24 ± 4.40 mm on the comparative side. In contrast, for participants in the Type II group (conventional MRONJ), peri‐implant osteonecrosis extended from extraction of adjacent teeth, showing a disseminated bone loss pattern without any noticeable trend (Figure 4). Thus, patients in the Type I group with osseointegrated prosthesis following implant placement had a higher probability of osteonecrosis in the main direction of the occlusal force compared with those in the Type II comparison group (OR, 12.67; *p < 0.001*) (Table 4).

**TABLE 4:** Bone loss tendency on main loading direction.

	Bone loss on main loading direction Numbers of implants (*n*)			OR (95% CI)
	Yes	No	Sum	
Type I. PI‐MRONJ	48	12	60	12.67 (4.16–38.62)
Type II. Conventional MRONJ*	6	19	25	Reference
Sum	54	31	85	< 0.001**

* For example, peri‐implant MRONJ extending from MRONJ from an adjacent tooth extraction site.

** *p*‐value: chi‐squared. OR, odds ratio; CI, confidence interval.

### 3.4 Lesion Diameter According to the Type of Implant‐Supported Crown Prostheses

Single implant‐supported crowns and implant‐supported multiunit fixed crowns (e.g., splinted, bridge prostheses) were examined under the assumption that as splinting disperses the force, a wider area would be affected by osteonecrosis in patients at risk of MRONJ. The cantilever implant fixed crown and implant‐supported or implant‐retained denture prosthetics and healing abutment after the implant placement were excluded.

**FIGURE 5:**
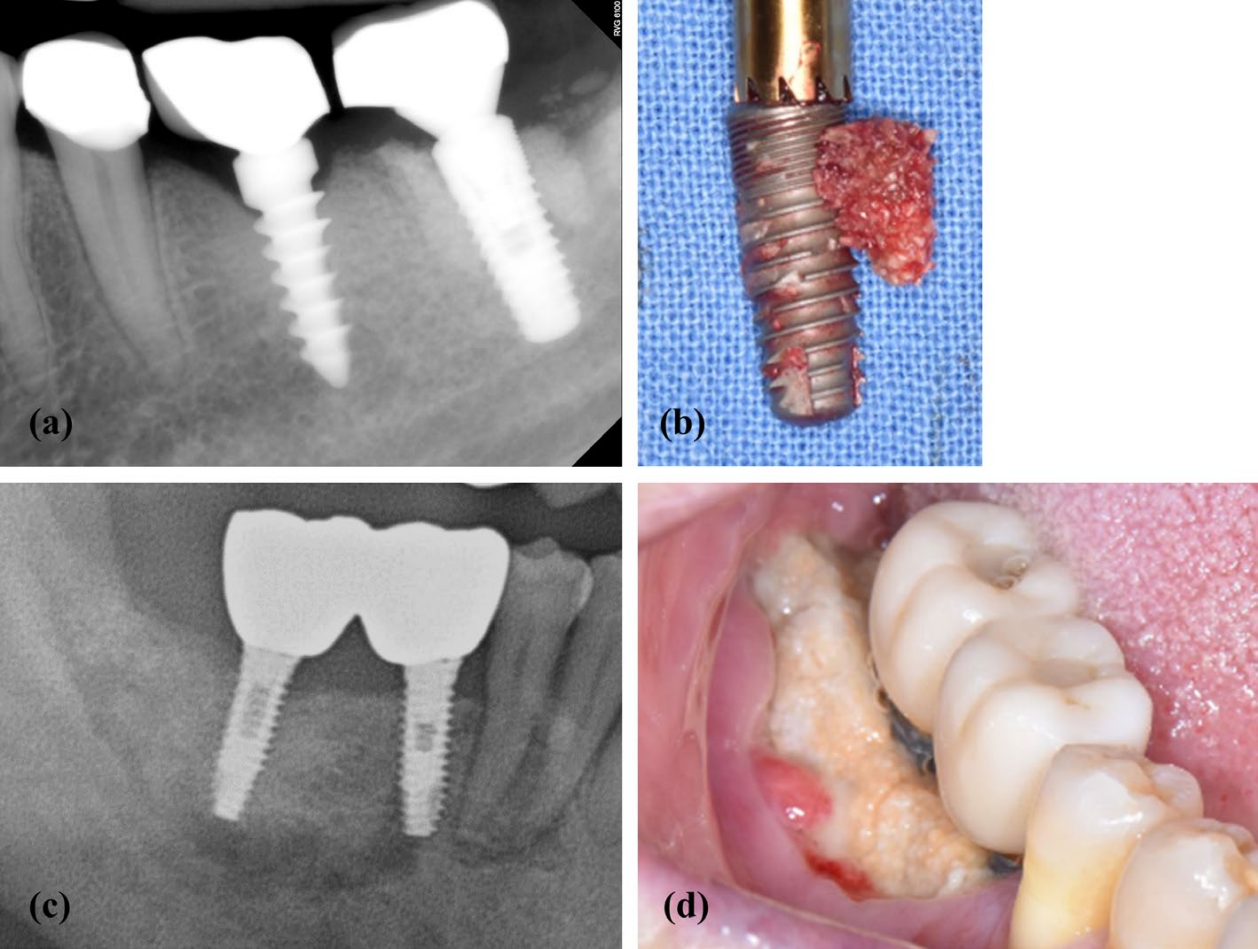
Affected lesion difference according to the type of prosthesis. (a) Preoperative radiographic view of a single implant supported crown. (b) Postoperative clinical photograph of a single implant supported crown peri‐implant medication‐related osteonecrosis of the jaw (PI‐MRONJ). (c, d) Preoperative radiographic view and clinical photograph of a splinted implant‐supported crowns.

The level of osteonecrosis in each patient was compared by measuring the length of the widest area on the CT axial view. In the 50 patients, the differences in PI‐MRONJ were compared on 13, 15, and 32 sites for the bridge, single, and splinted prostheses, respectively. The prevalence of lesions in each patient was analyzed as a continuous variable (Figure 5).

**FIGURE 6:**
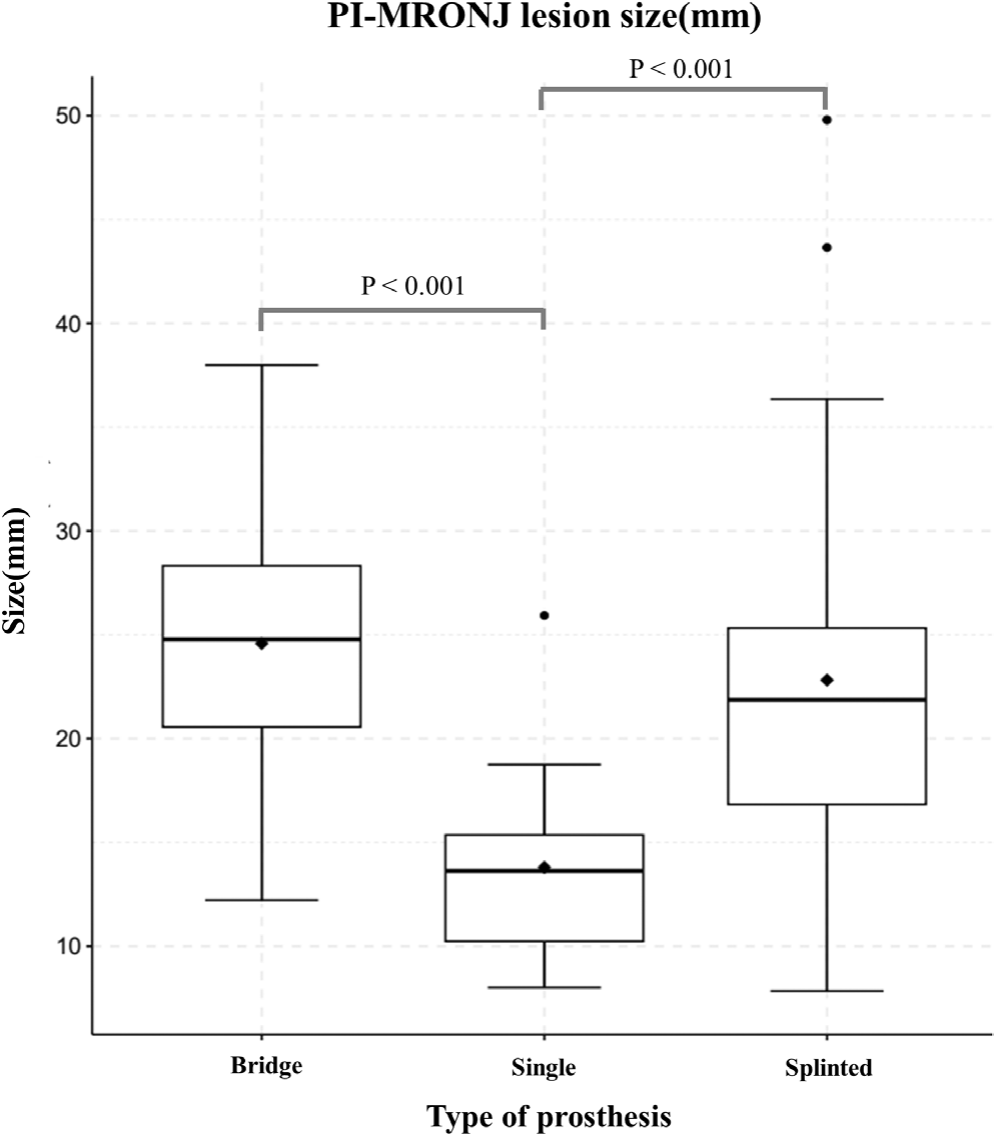
Boxplots representing lesion diameter according to the type of prosthesis.

The boundary of the area of osteonecrosis around the fixture was approximately 9.0 mm larger for the splinted implant‐supported crowns (22.82 mm) than for the single implant‐supported crown (13.80 mm). Thus, when the single vs. splinted and single vs. bridge prostheses were compared, the mean area of the lesion varied according to the prosthetic type (*p < 0.001*). However, when the bridge and splinted prostheses were compared, no significant between‐group difference was observed, as the two groups showed lesions of similar size (Figure 6).

## 4 Discussion

### 4.1 Principal Findings

We conducted a retrospective analysis of patients with PI‐MRONJ. For diagnosis, we evaluated the history of antiresorptive administration and osteonecrosis in the area around the implant in patients with a history of antiresorptive therapy for osteoporosis or cancer. Among those diagnosed with MRONJ, patients displaying clinically characteristic features of PI‐MRONJ were examined. The bone loss pattern was analyzed using radiographs and defined statistically. This single‐center retrospective study found that factors based on the implant inclination and type of prosthesis could impact the development and progression of PI‐MRONJ.

The direct causes of PI‐MRONJ are largely unknown at present. Nevertheless, various recent studies have reported their incidence after implant placement [10].

Owing to ethical considerations, there is a lack of randomized or prospective human trials on occlusal overload [11]. Clinically, the precise measurement of natural occlusal forces poses a challenge, hindering the identification of a clear correlation between occlusal forces (overload) and implant failure. Animal studies suggest that occlusal load can contribute to increased marginal bone loss around oral implants. While clinical observations also indicate heightened bone loss in high‐stress areas, a definitive causative link with overload has yet to be established. Nonaxial loading induces dynamic remodeling in the cortical and trabecular bone tissues, with observed osteoclastic activity in specific sites. Notably, nonaxial loads generate elevated stress levels in peri‐implant bone compared to axial loads [12].

Excessive biting force, resulting from decreased bone remodeling, may contribute to spontaneous MRONJ. Rats exposed to intravenous bisphosphonates exhibited increased microcrack, microinjury accumulation due to reduced bone remodeling capacity. These microcracks are suggested to compromise the biomechanical integrity of the jawbone, acting as potential sources for infection and subsequent osteonecrosis [13].

Zupancic Cepic et al. analyzed the stress distribution on the area around the implant fixture in relation to the type of prosthesis and reported that the splinted type showed more prominent global stiffness and axis loading than the single type but also showed a wider scope of stress distribution and a wider area for the oblique force [14]. As such, unlike the single implant‐supported crowns, the implant‐supported multiunit fixed crowns (e.g., splinted, bridge prostheses) dispersed the occlusal force across the area around the implant; however, its wider scope of action indicated that the lesion could extend to a wider area in patients at risk of MRONJ.

The observed trends may be attributed to issues in bone regeneration alongside bone destruction. This destruction is consistent with the dysfunction caused by reduced osteoclast activity, which is crucial for bone remodeling. This dysfunction is particularly evident in patients receiving antiresorptive drugs and it may also be influenced by the ongoing, lifelong remodeling of the jawbone. Furthermore, with sensitivity to mechanical loading, osteocytes synthesize various substrates in response to loading by expressing cytokines, nitric oxide, prostaglandin E2, and other growth factors that may exert substantial effects on peri‐implant bone metabolism [15, 16].

The pattern of peri‐implant bone loss was similar to that observed in the direction of the occlusal force in the area around the implant. Based on this finding, the variation based on the implant inclination was analyzed, and a significant correlation was found with the incidence of PI‐MRONJ.

This study also revealed a distinctive pattern of osteonecrosis development around the implant fixture. Unlike cases involving the extraction of an adjacent tooth, bone destruction was notably more pronounced in specific areas based on the implant inclination in the direction of nonaxial loading around the implant. This pattern persisted irrespective of the time of implant placement or the duration of antiresorptive administration.

### 4.2 Limitations of the Study and Future Recommendations

This study had certain limitations, such as a relatively small sample size. The sample is nonetheless considered representative and significant. Additionally, accurate analyses were not possible in some cases owing to the presence of metallic devices causing artifacts on radiographic images or the severity of peri‐implant bone loss. The method of measuring volume or area around the fixture may be relatively accurate; however, in this study, the method of measuring bone loss around the implant in other documents related to peri‐implantitis was first used. In this study, most patients underwent implantation with or without bone graft at other hospitals, which hindered determining the relationship between bone grafting and PI‐MRONJ incidence.

Of the total 124 implants in 75 patients, 60 implants affected by PI‐MRONJ were included in the statistical analysis. The GEE model was used under the assumption of no correlation structure (independence). Typically, when using the GEE model, a correlation between values is assumed, particularly in cases involving teeth and implant‐related data when multiple values are measured in one subject. The Intraclass Correlation Coefficient (ICC) was calculated to verify that there was no or weak correlation within the subject (independence). The ICC was 2.48 × e^−16^, which is very close to 0, indicating no correlation within the subject (independence).

When comparing the three prosthesis types, statistical analysis was performed using one‐way ANOVA. However, since some patients had multiple lesions, the groups examined were not entirely independent of each other.

Further studies should use animal models to monitor the progression of peri‐implant defects. This would allow histological and histomorphological analyses and research on the 3D fine elemental analysis of various implants. Furthermore, due to the low prevalence of PI‐MRONJ, further studies are warranted to determine differences in the antiresorptive dose or duration between the high‐ and low‐dose groups, and studies should take into account bone grafts in the surrounding environment and implant surfaces or product classifications according to the implant fixture itself.

Additional analyses of the correlations in patients on drugs such as ARDs that could induce osteonecrosis after implant placement would also be possible if occlusal force could be measured using methods such as T‐scans.

### 4.3 Clinical Implications

Medical factors, such as diabetes, corticosteroid use, and immunosuppressive therapy, and individual patient factors, such as smoking habits, chronic periodontal disease, and poor oral hygiene, are considered to affect MRONJ development [17]. The findings of this study implied that besides the soft‐tissue and inflammation‐related factors, such as periodontitis, that had been considered in previous studies [18], adequate consideration is required for factors related to alveolar bone and prostheses in the vicinity of PI‐MRONJ, whose cause remains unclear. A pattern of osteonecrosis without a notable trend was observed for MRONJ extending in the peri‐implant areas owing to the extraction of an adjacent tooth or other stimuli. In contrast, for implants facilitating mastication function through prostheses, bone loss predominantly occurred in specific peri‐implant areas, with a tendency to spread gradually in the surrounding region.

Currently, there is a paucity of studies specifically addressing the surgical treatment of PI‐MRONJ [19]. Resective surgery emerges as an effective approach for treating Stage II/III MRONJ owing to its limited morbidity, whereas conservative surgery exhibits less predictable outcomes. In a previous study, a surgical approach involving implant removal and bone debridement with sequestrectomy proved effective, resulting in complete healing in 86.7%. This is consistent with findings from other studies on MRONJ surgery [20]. Discontinuation of the current drug or administration of another drug should be considered through collaboration for internal medicine regarding drug administration. If periodic monitoring shows that the lesion has not extended further and is in an isolated state, early surgical intervention is recommended. We implemented a surgical treatment protocol for peri‐implant MRONJ, achieving a positive outcome in approximately 85% of cases.

Therefore, for patients using ARDs, it is necessary to understand their general condition before implantation and the ideal treatment plan. These patients require more regular follow‐up than other patients after implant prosthetic delivery.

## 5 Conclusions

In this study, novel clinical features of PI‐MRONJ were observed in patients taking ARDs. This indicates a heightened association with PI‐MRONJ occurrence and progression, specifically relative to implant inclination and prosthesis type in the posterior region. However, the need for a more controlled study to validate these clinical findings is emphasized. Preclinical experiments, including overload conditions and finite element analyses of bone microstrain, are essential to evaluate PI‐MRONJ robustly in future research.

## Author Contributions

H.‐G.J. was involved in data curation, formal analysis, investigation, methodology, visualization, and writing the original draft. W.P. was involved in data curation, methodology, validation, and reviewing and editing of the manuscript. I.‐H.C. was involved in data curation and formal analysis. Y.‐S.J. conceived of the study and was involved in funding acquisition, methodology, project administration, supervision, writing the original draft, and reviewing and editing the final manuscript. D.Y.L. was involved in data curation. J.‐Y.K. was involved in supervision, validation, and reviewing and editing the manuscript.

## Conflicts of Interest

The authors declare no conflicts of interest.

## Data Availability

The data that support the findings of this study are available on request from the corresponding author. The data are not publicly available due to privacy or ethical restrictions.
